# The Volatile Composition and the Potential Health Benefits of Different Microalgae Strains

**DOI:** 10.3390/foods13142174

**Published:** 2024-07-10

**Authors:** Madalena Grácio, Joana Ferreira, Pia Steinrücken, Dorinde M. M. Kleinegris, Isabel Sousa, M. Cristiana Nunes, Anabela Raymundo

**Affiliations:** 1 LEAF—Linking Landscape, Environment, Agriculture and Food—Research Center, Associate Laboratory TERRA, Instituto Superior de Agronomia, Universidade de Lisboa, Tapada da Ajuda, 1349-017 Lisboa, Portugal; madalenagracio@outlook.com (M.G.); isabelsousa@isa.ulisboa.pt (I.S.); cristiananunes@isa.ulisboa.pt (M.C.N.); anabraymundo@isa.ulisboa.pt (A.R.); 2 NORCE Norwegian Research Centre, Thormøhlensgate 53, 5006 Bergen, Norway; pias@norceresearch.no (P.S.); dokl@norceresearch.no (D.M.M.K.)

**Keywords:** microalgae, nutritional composition, antioxidant activity, antidiabetic activity, volatile organic compounds

## Abstract

The use of microalgae as a food ingredient has been gaining attention in recent years due to its nutritional benefits. The main goals of this study were to (i) assess the nutritional potential of *Chlorella vulgaris*, *Tetraselmis chuii*, *Microchloropsis gaditana*, and *Phaeodactylum tricornutum*; (ii) evaluate their bioactive properties (antioxidant activity, total phenolic content, and α-amylase inhibitory activity) and (iii) assess the main volatile compounds composition. The protein content was considerably high (32–44 mg/100 g dw) for all the microalgae strains. The DPPH scavenging potential range was 14–25 mg Trolox/100 g dw (highest for *T. chuii*) and the ferric reducing power ability range was 13–67 µmol Trolox/dw (higher for *T. chuii*). The total phenolic content range was 2–7 mg of gallic acid equivalents/g dw, for *M. gaditana* and *T. chuii*, respectively, which was mainly due to the presence of catechin (1–9 µg/g dw), epicatechin (3–29 µg/g dw), and vanillic acid (1–14 µg/g dw). The ɑ-amylase inhibitory potential range was 26–42%. *C. vulgaris* was richer in chlorophyll a (18 mg/g dw), whilst *T. chuii* was particularly rich in chlorophyll b (29 mg/g dw). *P. tricornutum* showed the highest carotenoid content (4 mg/g dw). Aldehydes and alkanes were the major compounds identified in *M. gaditana*, whereas alcohols and *N*-based compounds existed in higher amounts in *P. tricornutum*. *T. chuii* and *C. vulgaris* were enriched in ketones and alkenes. This study’s novelty lies in its comprehensive and integrative analysis of the nutritional, bioactive, and volatile properties of four distinct microalgae strains. By providing detailed comparisons and highlighting potential applications in functional foods, it offers a unique contribution to the field of microalgae research and its practical application in the food industry. This multifaceted approach sets it apart from existing studies, offering new insights and opportunities for leveraging microalgae as valuable food ingredients.

## 1. Introduction

Microalgae have emerged as a potent source of diverse phytochemicals with notable functional properties that can enhance human health. Their rich biochemical composition, which includes proteins, essential fatty acids, pigments, and antioxidants, offers substantial health benefits for humans [1].

Due to their impressive array of phytochemicals and nutritional value, microalgae are ideal candidates for inclusion in food products. This allows for the development of microalgae-based foods designed to offer functional benefits beyond basic nutrition [1]. Integrating microalgae into food formulations opens opportunities to deliver targeted health functions, contributing to overall well-being. However, this integration presents several challenges that need consideration. Technologically, adding microalgae can affect the food structure, altering rheological behavior and impacting texture and consistency [2]. Additionally, the inclusion of microalgal biomass may change the color and flavor profiles of food products, creating sensory challenges. Common issues include deviations from expected colors due to microalgal pigments and the potential for a fish-like odor from volatile compounds.

The aroma of microalgae arises from a variety of volatile organic compounds (VOCs), which are natural secondary metabolites produced by these organisms [3]. Identifying these VOCs is crucial for addressing sensory challenges and ensuring consumer acceptance. In this relatively unexplored area, specific volatile secondary metabolites might also possess significant bioactive properties. Therefore, the study focused on analyzing the compounds responsible for the overall aroma and investigating the potential bioactivity of volatile metabolites across various microalgae strains. This involved analyzing the volatile composition using GC-MS to establish a comprehensive database of microalgae VOC fingerprints. This detailed understanding of the influence of VOCs on microalgae aroma could reveal their bioactive potential. Additionally, the nutritional composition (moisture, ash, minerals, protein, total fat, total carbohydrates, carotenoids, chlorophylls a and b) and potential health benefits (antioxidant activity, phenolic content, α-amylase inhibition) of four microalgae species was assessed.

## 2. Materials and Methods

### 2.1. Material

Various freeze-dried strains of microalgae, including *C. vulgaris*, *T. chuii*, *P. tricornutum*, and *M. gaditana*, were cultivated at the NORCE Norwegian Research Centre in Bergen, Norway. The marine microalga *M. gaditana* CCMP526 was sourced from Bigelow NMCA, while *T. chuii* UTEX LB232 and *P. tricornutum* UTEX 640 were obtained from the UTEX culture collection. The freshwater microalga C. vulgaris (NIVA-CHL108) was acquired from the NIVA culture collection of Algae (NIVA-CCA).

Upon receiving cultures from their respective collections, stock cultures were maintained in 15 mL glass tubes under controlled conditions: 15 °C temperature and a 16:8 h light–dark cycle with a light intensity of 50 µmol m^−2^ s^−1^.

Cultivation of freshwater algae (*C. vulgaris*) was conducted using modified BBM medium, prepared with sterilized reverse osmosis water and enriched with specific nutrient concentrations. For marine algae (*P. tricornutum*, *M. gaditana*, and *T. chuii*), the NORCE medium, based on sterilized natural seawater with a salinity of 35 ppt, was utilized. Both media were enriched with a nutrient stock solution containing various micronutrients, including B, Cu, Fe, Mn, Mo, and Zn, chelated with DTPA, sourced from the fertilizer YaraTera REXOLIN APN.

### 2.2. Methods

#### 2.2.1. Culture Growth Conditions and Harvesting

For biomass production, cultures were up-scaled to four 300 mL bubble column photobioreactors (24 °C, 100 µmol m^−2^ s^−1^ for 24 h, infused with 0.2 μm filtered, 1% CO_2_ enriched air). The biomass was used to inoculate 25 L GemTube ^®^ tubular photobioreactors (PBR). For *T. chuii*, *P. tricornutum*, and *C. vulgaris*, biomass produced from the 25 L PBR was directly harvested, while for *M. gaditana*, the 25 L culture was subsequently taken to the National Algaepilot Mongstad. The 25 L PBR comprised a vertical tubular glass helix with 12 windings and an outside and inside diameter of 32 mm and 28 mm, respectively. The cultures were circulated through the helix by airflow (0.2 µm filtrated). The reactor was located in a climate-controlled room, where the temperature was kept at 23.0 ± 0.5 °C. The pH was kept between 7.8 and 8 through controlled pulse-wise sparging of 100% CO_2_ in the ingoing airstream. Artificial illumination was provided through LED light panels located outside the tubular helix set-up. The incident light intensity at the start of the cultivation was 30 µmol m^−2^ s^−1^ and was increased stepwise with increasing biomass concentration to 550 µmol m^−2^ s^−1^. Nitrate concentration was checked regularly with nitrate test strips (Quantofix), and when necessary, additional NaNO_3_ was added. Cultures were grown until an increase in biomass concentration leveled off at maximum light intensities (4–7 g L^−1^). For *T. chuii*, *P. tricornutum*, and *C. vulgaris*, the biomass from the 25 L photobioreactors were collected and dewatered by filtration (Vibro-lab3500, SANI membranes). The obtained slurry was subsampled in falcon tubes for trait analysis (3× frozen paste and 3× freeze-dried paste of approximately 1 g dried biomass). The remaining slurry was frozen, freeze-dried, and the obtained powder (75–200 g) was collected in plastic bags and stored at −20 °C until being shipped to Portugal.

At the National Algaepilot Mongstad, in Norway (60.8032° N, 5.0267° E), *M. gaditana* biomass was produced in a 250 L photobioreactor and four 750 L tubular photobioreactors (LGEM, The Netherlands), which were located in a greenhouse. Illumination was provided by natural light and artificial illumination (EAX 170W LED lights, Evolys AS, Norway) with an average of 220 µmol m^−2^ s^−1^. The reactors were operated at pH 7.8 by on-demand CO_2_ addition, and culture temperatures were maintained between 15 and 35 °C by cooling through heat exchangers located in the collection tanks of the PBRs, using circulating tap water, and heating through heaters inside the greenhouse. The reactors were operated in dual mode, as such mixing was provided by both liquid pump and air pump, resulting in a liquid velocity of approximately 0.3 m s^−1^. The algae were produced in a fed-batch process: the reactors were harvested twice per week (50–90% of the culture volume), after which seawater and nutrients were added to compensate for the volume taken. *M. gaditana* biomass was produced in various batches during April–July 2022. The biomass was harvested by dewatering using a spiral plate centrifuge (Evodos 25, Evodos BV, The Netherlands), resulting in a paste of approximately 25% dry weight (dw). The paste was vacuum packed and directly frozen at −20 °C before further processing. Some of the obtained paste was freeze dried (135 g powder) and subsampled for enzyme analysis. All samples were stored at −20 °C until being shipped to Portugal.

#### 2.2.2. Extraction

The extraction was performed in a Soxhlet apparatus for 3 h with ethanol (1:20 *w*/*v*) at reflux. The supernatant was collected and stored at −20 °C for further analyses (antioxidant activity, phenolic content, and α-amylase inhibition potential), and the pellet was dried and weighed.

#### 2.2.3. Nutritional Composition

The general nutritional composition included the determination of moisture, ash, minerals, protein, total fat, and total carbohydrates. The determination of each parameter was performed in triplicate, and the data were presented as mean ± SD. The moisture content of samples was measured gravimetrically through an automatic moisture analyzer PMB 202 (Adam Equipment, Oxford, NJ, USA) at 130 °C to a constant weight. The total ash content was determined by incineration at 500 °C in a muffle furnace [4]. Fat content was determined following the Portuguese standard method NP4168 [5]. The protein content (N × 6.25) was estimated by the combustion method DUMAS [6], using a Vario EL elemental analyzer (Elementar, Langenselbold, Germany). The carbohydrate content was calculated by the difference between the protein, fat, ash, and moisture contents.

#### 2.2.4. Quantification of Chlorophyll a, b, and Carotenoids

The methodology used to quantify chlorophylls and carotenoids was based on that used by Ah et al. [7]. Specifically, 0.5 µL of microalgae ethanolic extract (obtained as described in Section 2.2.2) was mixed with 5 mL of 96% (*v*/*v*) ethanol. Then, the absorbance at 470, 649, and 664 nm was read, and the quantification was carried out using the formula as follows:

(1) $${Ch}_{a\;}=(µg/mL)=13.36A_{664}-5.19A_{649}$$

(2) $${Ch}_{b\;\;}=(µg/mL)=27.43A_{649}-8.12A_{664}$$

(3) $$C_{a\;\;}=(µg/mL)=(1000A_{470}-2.13{Ch}_{a}-97.64{Ch}_{b})/209$$

#### 2.2.5. Antioxidant Activity

##### FRAP Assay

The FRAP assay followed the method of Benzie et al. [8]. The FRAP reagent was prepared by mixing 300 mM sodium acetate buffer (pH 3.6), 10 mM TPTZ (2,4,6-tri(2-pyridyl)-s-triazine; Sigma-Aldrich, ≥98.0% purity, St. Louis, MO, USA) solution, and 20.0 mM FeCl_3_·6H_2_O solution in a 10:1:1 (volume) ratio. An aliquot (90 μL) of either the extract or the standard was added to 3 mL of the FRAP reagent, and the mixture was incubated at 37 °C for 30 min. Absorbance was then measured at 593 nm against a blank. Calibration was performed using aqueous solutions of Trolox at known concentrations ranging from 0 to 700 µg mL⁻¹, yielding a linear calibration curve (y = 16.336x + 0.0432, r^2^ = 0.9997). Results were expressed as µmol Trolox equivalents per gram of dry mass.

##### DPPH Assay

The DPPH assay involved the use of 2,2-diphenyl-1-picrylhydrazyl hydrate (DPPH, Sigma-Aldrich, ≥99.0% purity, St. Louis, MO, USA), which is a nitrogen-centered free radical that changes color from purple to yellow when reduced by a radical scavenger, forming DPPH-H [9]. The results were reported as milligrams of Trolox equivalents per 100 g of dry weight (mg Trolox/100 g dw).

For the assay, different dilutions of the initial extract and a Trolox stock solution (Sigma-Aldrich, ≥97.0% purity, St. Louis, MO, USA) at 0.2 mg/mL in methanol were prepared. Each 100 μL aliquot of the methanolic solutions was mixed with 3.9 mL of DPPH solution in methanol (24 μg/mL). The blank consisted of 100 μL of methanol added to 3.9 mL of DPPH solution. Following a 40-min incubation at room temperature in the dark, absorbance was recorded at 515 nm.

The radical-scavenging activity of each sample was calculated by the DPPH inhibition percentage as follows: I % = [(Abs_A_ − Abs_sample_)/Abs_A_] × 100, where Abs_A_ was the absorbance of the blank and Abs_sample_ was the absorbance in the presence of the extract at different concentrations.

#### 2.2.6. Total Phenolic Content

The total phenolic content in the microalgae ethanolic extracts was assessed using a modified Folin–Ciocalteu method [10,11], which relies on the reduction of an ammonium molybdate complex by phenolics, producing blue-colored reaction products. Gallic acid (GA) served as the standard, and the experiment was performed in triplicate.

For the assay, a 150 μL aliquot of each extract, or the gallic acid standard, was combined with 140 μL of Folin–Ciocalteu reagent (diluted 1:10 *v*/*v*) and 2.4 mL of deionized water and vortexed. After 3 min at room temperature, 300 μL of sodium carbonate solution (7.5% *w*/*v*) was added, vortexed again, and the mixture was incubated at room temperature for 2 h in the dark. The absorbance of the resulting blue solutions was measured at 725 nm against a blank containing only water, using a UV-160A Recording Spectrophotometer (Shimadzu, Kyoto, Japan).

The same procedure was applied to prepare the gallic acid calibration curve (absorbance vs. [GA] mg/L), using seven separately prepared stock solutions of gallic acid at concentrations of 0, 50, 100, 150, 200, 250, and 350 mg/L (y = 33.632x + 0.0135, r^2^ = 0.9906). Absorbance was measured in triplicate at 725 nm. The total phenolic content was then expressed as mg of GA equivalent per gram of dry weight (mg GAE/g dw).

#### 2.2.7. Determination of Phenolic Compounds Composition

The extraction of phenolic compounds began by mixing 200 mg of biomass with 10 mL of methanol. After filtration, the filtrate was evaporated, and the dry residue was suspended in 5 mL of acidified water (pH 1.5). Solid-phase extraction (SPE) was then employed, following the procedure of Dvořáková et al. [12] with slight modifications. Chromabon Easy cartridges (Macherey-Nagel, 500 mg, particle size 93 μm) were conditioned by sequentially eluting with 2 mL of water, 6 mL of methanol, and 2 mL of water. The suspension passed through the cartridge, and the retained phenolics were eluted with 6 mL of acetone and then evaporated to dryness using a rotary vacuum evaporator. The residue was dissolved in 200 μL of the mobile phase B (methanol), filtered through a 0.20 μm nylon syringe filter, and transferred to a vial. Quantification was performed in triplicate.

Chromatographic analysis was conducted using a Vanquish Thermo Scientific HPLC system equipped with a quaternary pump (VC-P10-A), an autosampler (VC-A12-A), and a photodiode array detector (VC-D11-A). Data acquisition was managed with Chromeleon 6 software, version 6.40. Phenolics were separated using a Hypersil GOLD column (250 mm × 4.6 mm, 5 μm) with a guard column maintained at 20 °C. Gradient elution was carried out with water containing 1% acetic acid as mobile phase A and methanol as mobile phase B, using the following elution program for eluent A: 0–27 min, 90% isocratic; 27–28 min, linear gradient from 90% to 60%, then isocratic for 5 min; 34–36 min, gradient from 60% to 56%, followed by 8 min of isocratic elution; gradient from 56% to 90% over 1 min and reconditioning at 90% (isocratic for 4 min).

Simultaneous monitoring was set at 270 nm for gallic acid, catechin, vanillic acid, rutin, epicatechin, and syringic acid, and at 324 nm for coumaric acid and ferulic acid. Quantification was performed by injecting five different concentrations of each compound (ranging from 5 to 100 µg/mL) in triplicate. The presence of polyphenols in the extracts was confirmed by comparing their retention times and overlaying UV spectra with those of standard compounds.

#### 2.2.8. α-Amylase Inhibition

The α-amylase inhibition potential of the different microalgae strains under evaluation was determined following the method of Wickramaratne et al. [13] with some modifications: 200 µL of buffer (20 mM sodium phosphate buffer with 6.7 mM sodium chloride pH 6.9) and 200 µL of α-amylase (1 U/mL) were mixed with 200 µL of the samples by vortexing for 30 s and incubated in a water bath at 30 °C for 10 min. Afterward, 200 µL of the starch solution (1% *w*/*v* dissolved in the mentioned buffer) was added, and the samples were incubated in a water bath at 37 °C for 3 min. Then, 200 µL of 3,5-dinitro salicylic acid reagent (DNSA) was added, and the different samples were incubated in a water bath at 85 °C for 10 min. To finalize, the samples had to reach room temperature (around 24 °C), and the reaction was stopped by adding 5 mL of deionized water. The absorbances were measured at 540 nm. α-Amylase inhibition was calculated using the following formula: % of α-amylase inhibition = ((Abs_C+_ + Abs_sample_)/Abs_C+_) × 100, where Abs_C+_ is the absorbance of the positive control and Abs_sample_ is the absorbance of the sample.

#### 2.2.9. Volatile Organic Compounds Profile

Static headspace sampling was conducted using the TriPlus RSH System headspace autosampler (Thermo Finnigan, San Francisco, CA, USA). A 2.5 mL headspace syringe from the PAL System was employed for injecting 2 mL of sample from 20 mL headspace vials containing 1 g of measured dry sample. The autosampler settings were configured as follows: incubation temperature set to 80 °C; incubation time of 10 min; syringe temperature maintained at 100 °C; agitator speed set to 500 rpm; fill speed at 100 μL s^−1^; pullup delay of 1 s; injection speed at 500 μL s^−1^; pre- and post-injection delay of 500 ms; and flush time of 10 s. Post-injection, any carryover in the syringe was eliminated by an automatic flush with carrier gas.

Chromatographic separation was achieved using a Thermo Scientific TRACE 1300 gas chromatograph coupled with a Thermo ISQ mass selective detector. A DB-1 fused silica capillary column (30 m × 0.25 mm i.d., Agilent J & W Scientific, Santa Clara, CA, USA) with a 0.25 μm film thickness was utilized with helium serving as the carrier gas (purity > 99.9997 vol %) at a flow rate of 1.0 mL min^−1^. The oven temperature program was initiated at 60 °C (not held), followed by a linear temperature gradient at a rate of 3 °C min^−1^ until reaching a final temperature of 260 °C, which was held for 5 min (total run time: 65 min). The ion source temperature was maintained at 230 °C, the transfer line was maintained at 150 °C, and mass spectra were obtained in the 50 to 500 *m*/*z* range at an electron energy of 70 eV. Peak areas in the total ion chromatogram (TIC) were determined and expressed as normalized relative percentages. The calculated composition was semi-quantitative/qualitative as no standards for each chemical family were co-injected nor were their response factors determined. Each aliquot was injected in triplicate for robustness.

#### 2.2.10. Statistical Analysis

The one-way analysis of variance (ANOVA) was applied using XLSTAT Premium Statistical Software for Excel version 2021.4.1 (Addinsoft, New York, NY, USA) integrated with Microsoft Excel 2021 (Microsoft Corp., Redmond, WA, USA). A level of *p* ≤ 0.05 was considered significant.

## 3. Results

### 3.1. Nutritional Composition

**Figure 1:**
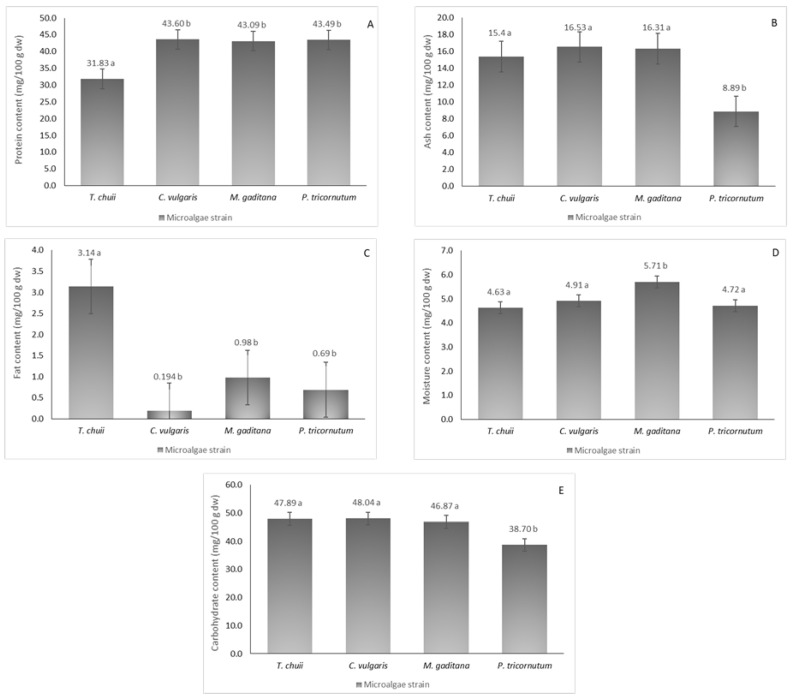
Nutritional composition of the different microalgae strains, *T. chuii*, *C. vulgaris*, *M. gaditana* and *P. tricornutum*. (**A**) Protein content, (**B**) ash content, (**C**) fat content, (**D**) moisture content, and (**E**) carbohydrate content. The results are expressed in mg/100 g of dried weight (dw) ± SD. The data shown are mean values (*n* = 3) followed by an alphabet letter. Different letters mean significant different results (Tukey’s HSD; *p* ≤ 0.05).

The nutritional profiles of the four different microalgae strains were analyzed, as illustrated in Figure 1, revealing considerable protein content ranging from 32 to 44 mg/100 g dw. Notably, *T. chuii* exhibited the lowest protein content, which was consistent with prior research [2]. In contrast, *M. gaditana* (43 mg/100 g dw), *C. vulgaris* (44 mg/100 g dw), and *P. tricornutum* (43 mg/100 g dw) showed higher protein content, aligning with existing literature [14], with no significant statistical differences between them (*p* ≤ 0.05).

Ash content, reflecting mineral concentration (Table 1), varied from 9 to 17 mg/100 g dw among the strains with *P. tricornutum* recording the lowest. Sodium levels were notably elevated across all strains, ranging from 1419 to 7299 mg/100 g dw, and particularly high in *T. chuii* and *C. vulgaris*, surpassing previous reports [15]. Conversely, *M. gaditana* and *P. tricornutum* exhibited lower sodium content than reported elsewhere [15]. Potassium emerged as the second most abundant mineral except for *C. vulgaris*, which significantly deviated from expected concentrations [16]. Calcium, phosphorus, magnesium, and sulfur displayed varying degrees of abundance across different strains.

Fat content was relatively modest, with *T. chuii* reaching 3 mg/100 g dw (*p* ≤ 0.05), while *P. tricornutum* and *C. vulgaris* showed lower content than previously reported [17]. Discrepancies may arise from differences in cultivation methods and lipid extraction processes. Moisture content ranged from 4 to 6 mg/100 g dw, underscoring the importance of reducing moisture levels for broader applications, as microalgal biomass typically contains up to 80% moisture. Carbohydrate content was notably high, ranging from 39 to 48.0 mg/100 g dw, which was consistent with literature findings for various microalgae species [18]. Overall, variations in mineral and fat composition compared to existing literature may be attributed to distinct growth conditions and extraction methodologies.

### 3.2. Chlorophyll and Carotenoid Quantification

**Figure 2:**
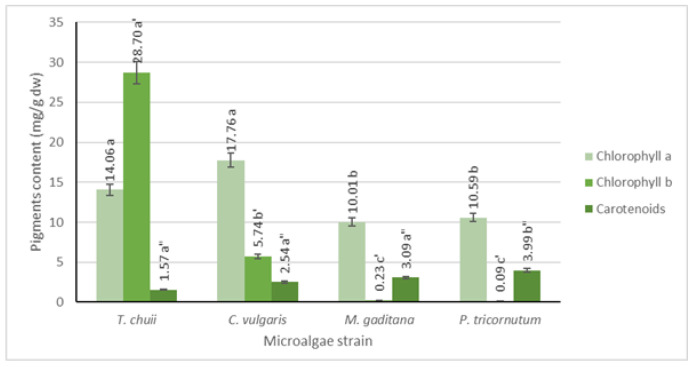
Quantification of chlorophyll a, b, and carotenoids for the different microalgae strains. The results are expressed in mg/g dw ± SD. The data shown are mean values (*n* = 3) followed by an alphabet letter. Different letters mean significant different results (Tukey’s HSD; *p* ≤ 0.05).

Pigment analysis was conducted using UV-Vis spectrophotometry, and the results are depicted in Figure 2. It is evident that all microalgae strains possess significant levels of chlorophyll a, ranging from 10 to 18 mg/g dw, with the highest concentration observed in *T. chuii* and *C. vulgaris*. Conversely, chlorophyll b is found in lower concentrations, except for *T. chuii*, where it reaches approximately 29 mg/g dw. Among photosynthetic organisms, chlorophyll a predominates, while chlorophyll b is the second most abundant pigment in plants and green microalgae, which is consistent with the obtained results [19]. Additionally, previous research indicates that *T. chuii* has a higher chlorophyll b concentration than chlorophyll a [20].

In terms of carotenoids, all microalgae strains exhibit lower levels compared to both types of chlorophyll (*p* ≤ 0.05), ranging from 2 to 4 mg/g dw, which was consistent with findings in the literature [21].

### 3.3. Antioxidant Activity and Total Phenolic Content

**Figure 3:**
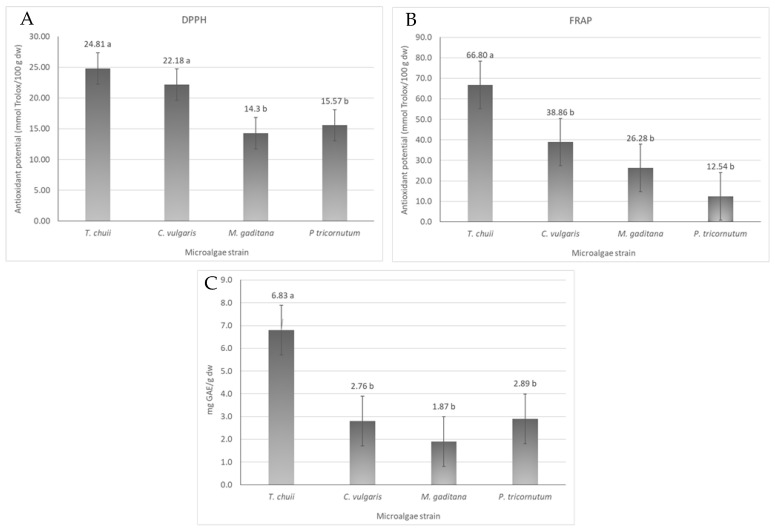
Antioxidant activity and total phenolic content of the different microalgae strains, *T. chuii*, *C. vulgaris*, *M. gaditana* and *P. tricornutum*. (**A**) Antioxidant potential measured by DPPH assay, expressed in mmol Trolox equivalents/100 g dw ± SD. (**B**) Antioxidant potential measured by FRAP assay, expressed in mmol Trolox equivalents/100 g dw ± SD. (**C**) Total phenolic content, expressed as mg GAE/g dw ± SD. The data shown are mean values (*n* = 3) followed by an alphabet letter. Different letters mean significant different results (Tukey’s HSD; *p* ≤ 0.05).

The antioxidant activity of different microalgae strains was evaluated by DPPH and FRAP assays. In addition, the total phenolic content of *T. chuii*, *C. vulgaris*, *M. gaditana* and *P. tricornutum* was assessed. The results are shown in Figure 3.

The DPPH scavenging potential of the microalgae ranged from 14 to 25 mmol Trolox/100 g dw in *M. gaditana* and *T. chuii*, respectively (Figure 3A). The antioxidant activity between *T. chuii* and *C. vulgaris* is not significantly different (*p* ≤ 0.05), being around 23 mmol Trolox/100 g dw, nor was the antioxidant activity of *M. gaditana* and *P. tricornutum* (*p* < 0.05), whose values were are around 15 mmol Trolox/100 g dw. In the FRAP assay (Figure 3B), the ferric-reducing power ability of the different microalgae ranged from 12 to 67 mmol Trolox/g dw, being higher for *T. chuii* and lower for *P. tricornutum*. These values are within the range of the expected antioxidant potential of microalgae with an extensive list of works reporting a wide array of antioxidant capacities [22] that will depend on the conditions used to growth the microalgal biomass, the harvesting conditions, and the microalgae strain itself [22].

Phenols constitute a critical group of natural compounds synthesized by microalgae as secondary metabolites. They exhibit diverse biological properties and play a pivotal role in safeguarding microalgae cells against both biotic and abiotic stressors [22]. However, the main biological activity associated with phenolic compounds is their antioxidant properties, which enable them to combat free radicals [22]. The types and amounts of phenolic compounds in microalgae can vary depending on factors such as the species, growth conditions, and extraction methods used. Some of the identified phenolic compounds in *T. chuii* include caffeic acid, ferulic acid, gallic acid, and p-coumaric acid [23]. In *Chlorella* spp. chlorogenic acid, caffeic acid, p-coumaric acid, vanillic acid, and syringic acid have also been identified [24]. *M. gaditana* is rich in gallic acid, catechin, epicatechin, and ferulic acid, and some phenolic compounds identified in *P. tricornutum* include protocatechuic acid, catechol, hydroquinone, and vanillic acid, among others [23].

The total phenolic content of the extracts of *T. chuii*, *C. vulgaris*, *M. gaditana*, and *P. tricornutum* was evaluated using the Folin–Ciocalteu method, and the results showed that the total phenolic content ranged from 2 to 7 mg of GAE/g dw, for *M. gaditana* and *T. chuii*, respectively (Figure 3C). In the literature, the variation in phenolic content is quite big, varying from 6 to 17 mg GAE/g dw for different solvent extracts of a wide variety of microalgae strains [25]. Based on the literature, the microalgae *T. chuii* yielded 7 GAE mg/g dw, which is consistent with values reported in previous studies 8–9 mg GAE/g dw [25]. Similarly, for *C. vulgaris*, a value of 3 GAE mg/g dw was obtained, as reported in the literature [26]. For *M. gaditana*, the results were also similar to what was previously reported [27]. Finally, for *P. tricornutum*, the results were also quite similar to those in the literature [28].

### 3.4. Identification and Quantification of Phenolic Compounds

The identification and quantification of eight polyphenols were achieved by the HPLC-DAD method adapted from Nour et al. [29]. The method linearity was evaluated using the method of least squares of a plot of integrated peak area versus mean concentration from three area measurements. The correlation coefficients were not less than 0.9995. Precision was assessed using six determinations at 1 μg mL^−1^ and expressed as relative standard deviation (RSD), ranging from 1.90 to 4.75%. The limits of detection (LODs) and the limits of quantification (LOQs) were calculated assuming minimum detectable signal-to-noise levels of 2 and 10, respectively. The LOD values were found to be in the range of 0.0233–0.2036 μg mL^−1^, and the LOQ values were observed in the range of 0.0649–0.5432 μg mL^−1^. The recoveries were found in the range from 85.3 to 99.7%.

Eight phenolic compounds were analyzed, but only four of them were identified for all the different microalgae strains (Table 2). Syringic acid and rutin were not detected in the extracts obtained from *T. chuii* and *P. tricornutum*. Syringic acid was also not detected in the extracts from *M. gaditana*, and ferulic acid was not identified in the extracts of *C. vulgaris* and *M. gaditana*. *C. vulgaris* exhibited the highest content of phenolic compounds (39.90 μg g^−1^ of dry weight) followed by *P. tricornutum* and *M. gaditana* (29.15 and 27.04 μg g^−1^ of dry weight), respectively.

### 3.5. α-Amylase Inhibition Potential

Recent research suggests that studies on the antidiabetic potential of microalgae are just emerging [1]. To evaluate the potential antidiabetic effects of the various microalgae strains, their ability to inhibit α-amylase activity was assessed. α-Amylase is an enzyme crucial for breaking down polysaccharides like starch or glycogen into maltose and dextrin molecules through the hydrolysis of α-D-(1→4) glycosidic bonds [13]. The antidiabetic activity was determined by evaluating the inhibitory potential of α-amylase.

**Figure 4:**
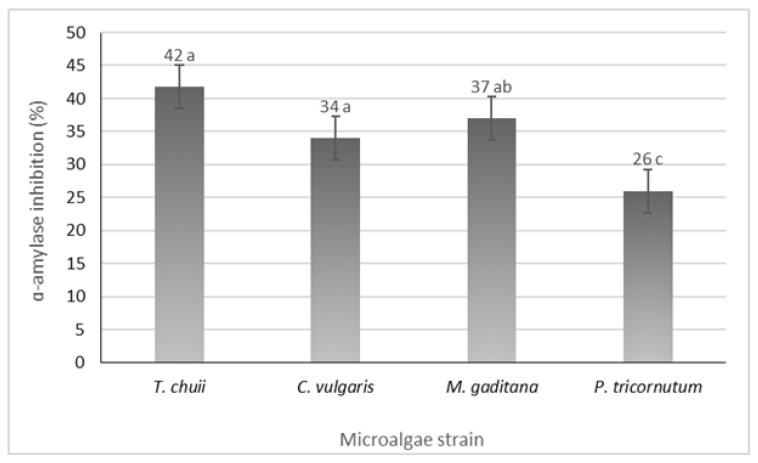
α-Amylase inhibition potential of the different microalgae strains. The results are expressed in % ± SD. The data shown are mean values (*n* = 3) followed by an alphabet letter. Different letters mean significant different results (Tukey’s HSD; *p* ≤ 0.05).

All the microalgae strains exhibited inhibitory activity, ranging from 26 to 42%, in *P. tricornutum* and *T. chuii* (Figure 4). Notably, microalgae such as *Nannochloropsis oculate* and *Arthrospira platensis* (Spirulina) have demonstrated significant antidiabetic activity through α-amylase inhibition with inhibition rates of 78% and 96%, respectively [30]. Additionally, incorporating *M. gaditana* into the diets of streptozotocin (STZ)-induced diabetic rats has shown antidiabetic activity by reducing blood glucose levels and glycated hemoglobin [13]. The observed α-amylase inhibition could be a key factor contributing to these results. According to these findings, different strains of microalgae have the potential to serve as a valuable resource in managing diabetes.

### 3.6. Volatile Organic Compounds Profile

Volatiles in food play a crucial role in the sensory experience of eating. They contribute to the flavor, texture, and overall appeal of food [3]. The results for the volatile organic compounds composition obtained by GC–MS analysis are summarized in Table 3. The main volatiles are aldehydes (5–17%, in *M. gaditana* and *C. vulgaris*, respectively), alcohols (7–31% of all compounds in *T. chuii* and *P. tricornutum*, respectively), ketones (2–23% of all compounds in *M. gaditana* and *T. chuii*, respectively), alkanes (1–58% of all compounds in *T. chuii* and *M. gaditana*), alkenes, and alkynes (0–22% of all compounds in *T. chuii* and *C. vulgaris*, respectively). *N*-based compounds were also identified in the different microalgae strains ranging from 1% to 48% in *M. gaditana* and *T. chuii*, respectively. Terpenoids represent 2–13% of all compounds in *M. gaditana* and in *P. tricornutum*. 2-Pentylfuran was also identified in smaller amounts (1–4%).

In the microalgae strain *T. chuii*, *N*-based compounds dominate, constituting 48% of the total volatiles. The major *N*-based volatiles are 2,4,5,6-tetramethylpyrimidine (18%), which is followed by 2,5-dimethylpyrazine (11%) and 2-ethyl-5-methylpyrazine (10%). Ketones are another significant class, comprising 23% of all volatiles, with 6-methyl-5-hepten-2-one being particularly prominent, making up 76% of this category. Aldehydes account for 10%, terpenoids for 7%, alcohols for 7%, and *S*-based compounds for 3%. Alkanes, alkenes, and alkynes collectively make up less than 2% of *T. chuii*’s volatile profile. Notably, the volatile composition in *T. chuii* varies significantly with growth conditions, harvesting, and storage practices. Some studies highlight α-ionone and β-ionone as the predominant terpenes, while others suggest aldehydes, ketones, and alcohols are more common, with *N*-based compounds being less prevalent. In *C. vulgaris*, the alkene 8-heptadecene is the most abundant volatile, constituting 22% of the total. Aldehydes are also significant, representing 17%, with 1-hexanal being the most prominent, accounting for 26% of the aldehyde fraction. Alkanes form the second most abundant class with nonadecane and pentadecane contributing 63% and 29% of all alkanes, respectively. Alkynes and alcohols collectively make up around 24% of the volatiles. *N*-based compounds account for 9% with 2,5-dimethylpyrazine being the most abundant *N*-based volatile (44%). Additionally, 2-pentylfuran is present at 4%. Some researchers identify aldehydes and S-based compounds as the major volatiles in *C. vulgaris*. *M. gaditana* is particularly rich in alkanes, which constitute 58% of its volatile profile, with pentadecane (35%) and nonadecane (11%) being the dominant alkanes, together making up 92% of the alkane fraction. Alkenes and alcohols together account for 22%, with alcohols being the third most abundant class at 16%. Aldehydes and ketones each represent 7% of the total, while terpenoids, *S*-based, and *N*-based compounds each constitute 4%. Previous reports frequently identify β-cyclocitral and various alkenes as major volatiles in *M. gaditana*. In *P. tricornutum*, alcohols and *N*-based compounds are the predominant classes, comprising around 63% of the total volatiles, with 2,5-dimethylpyrazine and 4,5,6-tetramethylpyrimidine being the major constituents (22% combined). Terpenoids account for 13%, with α- and β-ionones representing 53% of this category. Aldehydes are also present in significant amounts (9%), with benzaldehyde being the most representative (66% of the aldehydes). Alkanes and ketones are found in smaller amounts, at 5% and 4%, respectively. Some studies report that alkenes, such as 6-((E)-butenyl)-1,4-cycloheptadiene, are major volatiles in *P. tricornutum*.

Analyzing these results, and comparing them to the most recent literature [3], hundreds of volatiles can contribute to the flavor of microalgae, including terpenes, aldehydes, alcohols, ketones, nitrogen compounds, and more.

Both the study and literature emphasize the significant role of aldehydes in the aroma profile of microalgae. The range observed in the study (5–17%) aligns with literature reports that aldehydes are critical due to their low odor thresholds and varied odors [3]. Concerning alcohols, the study highlights them as major components (7–31%), particularly in *P. tricornutum*. This is consistent with literature, which notes that alcohols, especially branched-chain varieties, contribute to fruity and pleasant odors [3]. The presence of ketones (2–23%) in the study matches the literature’s description of their significant olfactory impact, though the odor can range from mushroom-like to bland, depending on the ketone’s structure [3]. In addition, the study identifies N-based compounds in all strains, particularly in *T. chuii* (48%), agreeing with literature findings that nitrogen compounds, while less common, can significantly affect the aroma, often contributing to off-flavors [3]. Representing 2–13% in the study strains, terpenoids are crucial for floral and woody scents, as noted in the literature. Their role in aroma is well supported, particularly for those like α- and β-ionones identified in *P. tricornutum* [3].

The analysis of volatiles in these four microalgae strains underscores their diverse and complex flavor profiles. While the specific volatile compositions vary among strains, they collectively align with the broader understanding from the literature that microalgae’s aroma and flavor are influenced by a myriad of volatile compounds, each contributing differently to the sensory experience. Understanding these profiles can help optimize the use of microalgae in food products, ensuring desirable sensory qualities and consumer acceptance.

## 4. Conclusions

By embracing microalgae as a sustainable protein source, we have the opportunity to pave the way for a more environmentally friendly and resilient food production system. This transition toward innovative and eco-conscious practices holds promise for securing a better future for both humanity and the planet. Furthermore, microalgae boast bioactive compounds with notable health-promoting properties. Their potent antioxidant activity and ability to inhibit the α-amylase enzyme position them as potential agents in the fight against diabetes. The combination of their nutritional richness and the presence of bioactive compounds renders microalgae an exceptional candidate for the food industry not only as a protein source but also for creating functional food products.

Based on the study’s findings, we conclude with an analysis of which microalgae might be recommended for incorporation into food formulations and as a food supplement, considering aspects such as color, smell, taste, and VOCs. Overall, *T. chuii* and *C. vulgaris* are recommended for food formulations due to their balanced nutritional benefits, bioactive properties, and more favorable VOC profiles. *T. chuii* is particularly noted for its antioxidant capacity and phenolic content, making it a strong candidate as a functional food supplement. The VOC profiles suggest that while some algae like *M. gaditana* and *P. tricornutum* may offer significant nutritional benefits, their aroma may pose challenges for broader food applications.

## Figures and Tables

**Table 1:** Mineral composition of the microalgae *T. chuii*, *C. vulgaris*, *M. gaditana,* and *P. tricornutum*. The results are expressed in mg/100 g of dry weight (dw) ± SD. The data shown are mean values (*n* = 3) ± SD followed by an alphabet letter. Different letters mean significant different results (Tukey’s HSD; *p* ≤ 0.05).

Sample	*T. chuii*	*C. vulgaris*	*M. gaditana*	*P. tricornutum*
Na	6652.1 ± 87.16 ^a^	1610.4 ± 45.13 ^b^	1418.9 ± 17.91 ^d^	7299.0 ± 211.96 ^e^
K	1227.1 ± 21.56 ^a^	374.5 ± 32.85 ^b^	1239.9 ± 97.33 ^d^	2532.1 ± 135.63 ^e^
Ca	2273.3 ± 238.36 ^a^	65.5 ± 1.69 ^b^	196.5 ± 41.63 ^d^	457.9 ± 82.13 ^e^
Mg	822.6 ± 42.33 ^a^	105.1 ± 23.77 ^b^	493.7 ± 17.87 ^d^	994.4 ± 63.79 ^e^
P	299.0 ± 47.13 ^a^	786.7 ± 6.90 ^b^	1036.5 ± 26.47 ^d^	562.1 ± 74.30 ^e^
S	888.3 ± 32.71 ^a^	224.7 ± 21.88 ^b^	108.8 ± 18.03 ^d^	819.6 ± 83.3 ^e^
Fe	nd ^1^	18.1 ± 0.63 ^a^	36.6 ± 14.72 ^c^	8.2 ± 0.04 ^f^
Mn	nd ^1^	nd ^1^	nd ^1^	nd ^1^

^1^ nd—non-detected.

**Table 2:** Total polyphenol contents (µg g^−1^ dw ± SD) of the analyzed microalgae strains. The data shown are mean values (*n* = 3) ± SD followed by an alphabet letter. Different letters mean significant different results (Tukey’s HSD; *p* ≤ 0.05).

Polyphenol	*T. chuii*	*C. vulgaris*	*M. gaditana*	*P. tricornutum*
Gallic acid	1.04 ± 0.034 ^a^	2.21 ± 0.140 ^b^	6.47 ± 0.560 ^c^	1.19 ± 0.103 ^a^
Caffeic acid	1.16 ± 0.052 ^a^	nd	0.47 ± 0.000	1.10 ± 0.002
Catechin	8.76 ± 0.400 ^a^	6.29 ± 0.302 ^b^	1.14 ± 0.113 ^c^	3.27 ± 0.007 ^d^
Epicatechin	3.01 ± 0.741 ^a^	28.71 ± 1.01 ^b^	3.15 ± 0.805 ^c^	12.56 ± 1.194 ^d^
Vanillic acid	1.31 ± 0.013 ^a^	1.16 ± 0.044 ^b^	14.16 ± 0.471 ^c^	10.14 ± 1.022 ^d^
Syringic acid	nd ^1^	0.74 ± 0.012 ^a^	nd ^1^	nd ^1^
Rutin	nd ^1^	0.79 ± 0.010 ^a^	1.65 ± 0.023 ^b^	nd ^1^
Ferulic acid	0.11 ± 0.004 ^a^	nd ^1^	nd ^1^	0.89 ± 0.007 ^b^
Total	15.35	39.90	27.04	29.15

^1^ nd—non-detected.

**Table 3:** Volatile lipophilic compounds identified in *T. chuii*, *C. vulgaris*, *M. gaditana,* and *P. tricornutum*, in % ± SD of the normalized peak areas in TIC. The data shown are mean values (n = 3).

*Compound*	*T. chuii*	*C. vulgaris*	*M. gaditana*	*P. tricornutum*
Aldehydes	10.40 ± 1.032	17.20 ± 1.284	5.06 ± 1.351	8.63 ±2.033
hexanal	2.22 ± 0.001	4.40 ± 0.004	-	-
heptanal	-	1.76 ± 0.001	-	1.59 ± 0.002
decanal	0.11 ± 0.002	0.67 ± 0.003	0.84 ± 0.044	0.57 ± 0.001
valeraldehyde	2.22 ± 0.007	0.97 ± 0.000	0.17 ± 0.000	-
benzaldehyde	2.01 ± 0.013	1.82 ± 0.010	3.71 ± 0.007	5.68 ± 0.002
9,12-octadecadienal	0.21 ± 0.004	0.73 ± 0.003	-	0.34 ± 0.001
2,6,6-trimethyl-1,3-cyclohexadiene-1-carboxaldehyde	1.43 ± 0.001	1.46 ± 0.003	0.34 ± 0.016	0.45 ± 0.051
Alcohols	6.71 ± 0.801	11.47 ± 1.663	10.96 ± 2.033	31.41 ± 3.498
benzenemethanol	2.27 ± 0.021	1.40 ± 0.107	0.67 ± 0.004	5.24 ± 0.102
2-ethyl-1-hexanol	-	0.49 ± 0.004	1.85 ± 0.033	5.11 ± 0.103
6-methyl-5-hepten-2-ol	-	3.34 ± 0.016	0.84 ± 0.045	10.17 ± 0.400
1-undecanol	0.74 ± 0.004	4.31 ± 0.214	-	1.02 ± 0.001
1-tetradecanol	-	0.24 ± 0.004	0.84 ± 0.074	2.16 ± 0.001
1-nonanol	0.37 ± 0.013	0.06 ± 0.017	-	1.48 ± 0.004
2-hexadecanol	1.06 ± 0.006	0.24 ± 0.003	-	-
1-hexadecanol	-	-	6.07 ± 0.142	-
Ketones	22.92 ± 9.475	2.25 ± 0.559	2.02 ± 0.955	4.09 ± 0.481
6-methyl-5-hepten-2-one	18.16 ± 0.211	1.52 ± 0.097	1.69 ± 0.044	1.70 ± 0.411
nona-3,5-dien-2-one	4.76 ± 0.143	0.73 ± 0.007	0.34 ± 0.021	2.38 ± 0.063
Alkanes	1.16 ± 0.223	16.75 ± 4.001	57.62 ± 15.575	5.56 ± 1.340
pentadecane	0.21 ± 0.001	4.92 ± 0.031	34.69 ± 7.085	0.57 ± 0.102
dodecane	0.58 ± 0.340	0.24 ± 0.002	1.52 ± 0.021	3.29 ± 0.501
nonadecane	0.32 ± 0.047	10.56 ± 1.041	18.55 ± 2.140	1.36 ± 0.320
docosane	0.05 ± 0.004	1.03 ± 0.002	2.87 ± 0.047	0.34 ± 0.000
Alkenes	0.16 ± 0.031	22.09 ± 3.458	10.62 ± 0.320	0.68 ± 0.000
8-heptadecene	0.16 ± 0.031	22.09 ± 3.458	10.62 ± 0.320	0.68 ±0.000
Alkynes	0.05 ± 0.000	11.96 ± 0.901	0.34 ± 0.041	-
7-octadecyne, 2-methyl	0.05 ± 0.000	11.96 ± 0.901	0.34 ± 0.041	-
S-based compounds	3.01 ± 0.014	4.19 ± 0.307	0.67 ± 0.000	-
2-pentylfuran	3.01 ± 0.014	4.19 ± 0.307	0.67 ± 0.000	-
N-based compounds	47.53 ± 6.575	9.41 ± 1.605	1.01 ± 0.198	32.37 ± 4.752
2-methylpyrazine	-	3.28 ± 0.001	0.34 ± 0.103	-
2,5-dimethylpyrazine	10.83 ± 0.036	4.19 ± 0.474	-	12.39 ± 2.166
2-ethyl-5-methylpyrazine	9.72 ± 0.302	0.55 ± 0.025	0.51 ± 0.007	6.47 ± 1.252
2,5-dimethyl-3-ethyl-pyrazine	5.65 ± 0.432	0.49 ± 0.002	-	2.50 ± 0.020
2,4,5,6-tetramethylpyrimidine	18.22 ± 1.004	0.30 ± 0.201	0.17 ± 0.022	9.19 ± 1.140
4-ethyl-2,5,6-trimethylpyrimidine	3.06 ± 0.026	0.06 ± 0.000	-	1.70 ± 0.002
1,2-dihydro-1,4-diphenylphtalazine	-	0.24 ± 0.041	-	-
cathine	0.05 ± 0.000	0.30 ± 0.004	-	0.11 ± 0.004
Terpenoids	6.92 ± 0.889	2.06 ± 0.358	1.69 ± 0.266	12.94 ± 2.123
nerolidol	-	-	0.34 ± 0.003	-
b-cyclocitral	1.48 ± 0.003	0.97 ± 0.012	0.51 ± 0.007	1.70 ± 0.031
a-ionone	2.43 ± 0.122	0.30 ± 0.000	-	5.11 ± 0.007
b-lonol	1.27 ± 0.047	0.30 ± 0.001	0.17 ± 0.000	1.70 ± 0.611
b-ionone	1.74 ± 0.003	0.49 ± 0.025	0.67 ± 0.120	4.43 ± 0.102
Other	0.63 ± 0.445	0.42 ± 0.127	0.67 ± 0.474	0.57 ± 0.403
guanidinosuccinimide	0.63 ± 0.445	0.30 ± 0.007	-	0.57 ± 0.403
2’-o-methylguanosine	-	0.12 ± 0.041	0.67 ± 0.474	-
Total identified compounds	99.40 ± 14.931	97.8 ± 7.439	90.66 ± 17.530	96.25 ± 12.465
Non-identified compounds	0.51	2.20	9.34	3.75
Total	100.00	100.00	100.00	100.00

## Data Availability

The original contributions presented in the study are included in the article; further inquiries can be directed to the corresponding author.
